# Sports-Related Pediatric Facial Trauma: Analysis of Facial Fracture Pattern and Concomitant Injuries

**DOI:** 10.1055/s-0039-1697627

**Published:** 2019-10-09

**Authors:** Andrew A. Dobitsch, Nicholas C. Oleck, Farrah C. Liu, Jordan N. Halsey, Ian C. Hoppe, Edward S. Lee, Mark S. Granick

**Affiliations:** 1 Division of Plastic and Reconstructive Surgery, Department of Surgery, Rutgers New Jersey Medical School, Newark, New Jersey

**Keywords:** facial fracture, sports facial fracture, pediatric facial fracture, pediatric facial trauma

## Abstract

**Objective** Sports-related injuries, such as facial fractures, are potentially debilitating and may lead to long-term functional and aesthetic deficits in a pediatric patient. In this study, we analyze sports-related facial fractures in the urban pediatric population in an effort to characterize patterns of injury and improve management strategies and outcomes.

**Methods** Retrospective chart review was performed for all facial fractures resulting from sports injuries in the pediatric population at a level-1 trauma center (University Hospital, Newark, NJ).

**Results** Seventeen pediatric patients were identified as having sustained a fracture of the facial skeleton due to sports injury. Mean age was 13.9 years old. A total of 29 fractures were identified. Most common fracture sites included the orbit ( *n* = 12), mandible ( *n* = 5), nasal bone ( *n* = 5), and zygomaticomaxillary complex ( *n* = 3). The most common concomitant injuries included skull fracture ( *n* = 3), intracranial hemorrhage ( *n* = 4), and traumatic brain injury ( *n* = 4). One patient was intubated upon arrival to the emergency department. Hospital admission was required in 13 patients, 4 of which were admitted to an intensive care setting. Nine patients required operative intervention. Mean length of hospital stay was 2.4 days. No patients were expired.

**Conclusions** Sports-related facial fractures are potentially debilitating injuries in the pediatric population. Analysis of fracture pattern and concomitant injuries is imperative to develop effective management strategies and prevention techniques.

Each year, sports-related injuries account for 3 to 29% of facial injuries and 10 to 42% of facial fractures in the United States pediatric population. 1 With 66% of boys and 52% of girls ages between 5 and 18 years, participating in organized sports annually, a significant proportion of the population is at risk for these injuries. 2 While improvements in prevention techniques and safety equipment has led to a decrease in the overall number of pediatric facial fractures, they remain a significant cause of facial trauma in this demography. 1 As high-risk and high-impact sporting activities continue to grow in popularity, additional studies are required to assess these injures and improve safety protocols.

All contact sports pose a potential risk of facial fracture, but specific sports have been shown to pose a significantly greater risk. A recent study found that injuries sustained, while playing baseball or softball were significantly more likely to involve facial fractures, as opposed to injuries resulting from football or basketball related activities. 3 Investigation of the different patterns and mechanisms of injury seen in individual sports may aid health care providers in making diagnostic decisions and choosing management strategies.

Facial fractures and concomitant injuries of the developing pediatric facial skeleton can have long-term implications; therefore, accurate diagnosis and treatment are vital. 4 Pediatric patients have less calcified facial bones and a higher cranial–to-facial ratio than adults, thus growth potential must be addressed when considering different management strategies. 5 Due to the effect of facial trauma and surgical intervention on future development, these injuries need to be followed until bone maturation is complete. 5

Children are less prone to facial fractures than adults due to certain anatomical attributes and account for only 15% of facial fractures. 6 However, when they do occur, they tend to have severe concomitant injuries due to the high threshold of force required for injury. 6 These injuries include traumatic brain injury, intracranial hemorrhage, and severe head and chest trauma. 4 The relatively low prevalence of pediatric facial fractures has led to an incomplete assessment of these injuries in the current literature. Further investigation is required to accurately categorize patterns of injury, improve long-term outcomes, and develop targeted safety and prevention measures. In this study, the authors examine our experience with pediatric facial fractures secondary to sports-related injuries at an urban level-1 trauma center.

## Methods

A retrospective chart review was performed for all facial fractures in a level-1 trauma center in an urban environment (University Hospital, Newark, NJ) from 2002 to 2012 based on International Classification of Disease, revision 9 (ICD-9) codes. Facial fractures were diagnosed radiologically via facial computed tomography (CT) scans, magnetic resonance imaging (MRI), and plain X-ray films. All cases of facial fracture in the pediatric population, defined as ≤18 years of age, with sports-related injuries, as the modality of injury were included. Chart review was performed for each case that fit these predefined criteria. Information collected included patient demographics, fractures site, concomitant injuries, length of hospital stay, critical complications, and surgical management strategies. Final data were recorded on an Excel spreadsheet, and relevant statistical tests including Pearson's *χ* ^2^ test and odds ratio were performed, with the *p* < 0.05 set as the degree of statistical significance.

## Results

During the time period examined, 17 patients met inclusion criteria for our study. The mean age was 13.9 (range, 9–18) years, with a male predominance of 82.4%. Caucasians comprised of 41.2%, African American and Hispanic both accounted for 17.65%, and the remaining 23.5% were listed as other.

**Fig. 1:**
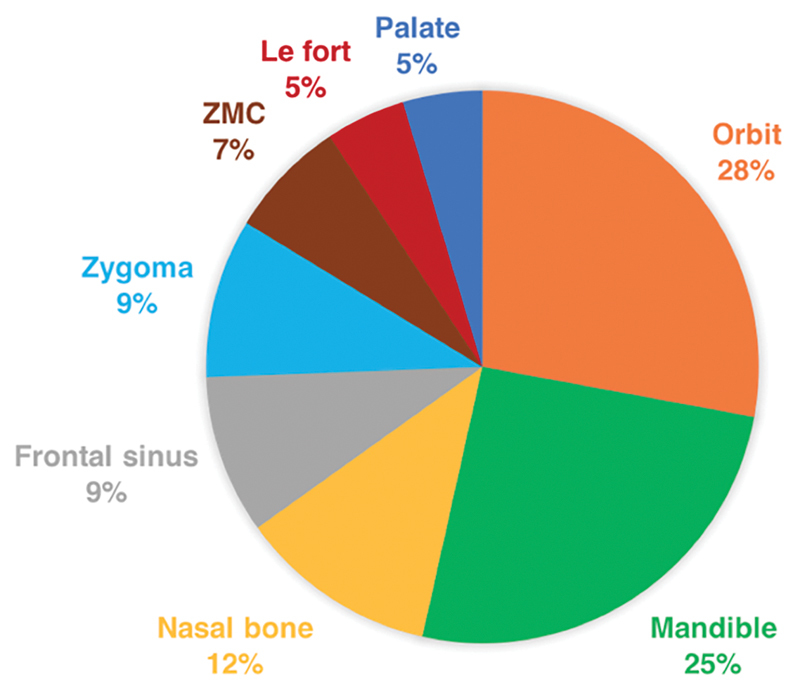
Distribution of facial fractures by anatomical site. ZMC, zygomaticomaxillary complex.

A total of 45 fractures were identified on radiologic imaging via CT scan, MRI, or plain X-ray films. The most common fractures were those of the orbit (28%), mandible (25%), and nasal bone (12%). Distribution of fractures by anatomical sites is demonstrated in Fig. 1 . One patient was intubated on, or prior to, arrival to the trauma bay. The mean hospital length of stay was 2.4 (range, 0–14) days. Four patients were admitted to the intensive care unit (ICU). No patients expired in our series. There was one patient who had an isolated skull fracture; however, the patient was not included because he failed to meet our criteria.

Three patients had concomitant intracranial hemorrhage. This was further broken down by intraparenchymal, subarachnoid, and subdural hemorrhage, and is displayed in Table 1 . One patient suffered a midline shift. Skull fractures were present in two patients and traumatic brain injury in four patients. Surgical management was required in 9 of the 17 patients, with a mean operative time of 189.9 (range, 93–440) minutes. All patients who required operative intervention underwent open reduction and internal fixation. Maxillomandibular fixation with arch bars were implemented in two patients, while three required Medpor implants, and four required titanium plates ( Table 2 ).

**Table 1:** Summary of pediatric facial fracture patients with sports-related trauma as the mechanism of injury

	Count (%)
Patients	17
Age in years (mean)	9–18 (13.9)
Gender	
Male	82.4
Female	17.6
Nationality	
African American	17.65
Hispanic	17.65
White	41.2
Other	23.5
Intubated	1
Hospital stay in days (mean)	0–14 (2.4)
Admissions to ICU	4
Fatality	0
Surgical management	9
Operative time, min (mean)	93–440 (189.9)

Abbreviation: ICU, intensive care unit.

**Table 2:** Summary of surgical procedures on pediatric patients with sports-related facial fractures

Age (y)/sex	Surgical procedure	MMF/arch bars	Medpor implant	Titanium plate
14/M	ORIF of right ZMC	0	1	1
14/M	ORIF of right ZMC	0	0	1
14/M	ORIF of left parasymphysis and right mandibular angle Surgical extraction of tooth no. 32	0	0	0
15/M	ORIF of left frontal sinus and anterior nasomaxillary region Closed reduction of bilateral nasal bone fractures	0	0	1
15/M	ORIF of left orbital floor	0	1	0
15/M	ORIF of left angle of mandible	1	0	0
15/M	ORIF of right orbital floor	0	1	0
18/M	ORIF of right parasymphysis of mandible	0	0	0
18/M	ORIF of Lefort	1	0	1

Abbreviations: F, female; M, male; ORIF, open reduction and internal fixation; ZMC, zygomaticomaxillary complex.

## Discussion

### Demographics

In the included cases in this study, we identified a mean patient age of 13.9 years, and the youngest included patient was represented by a 9-year-old male. These age disparities seen, and the lack of younger aged children, may be due to an increase in participation in contact sports, as children develop into adolescents. It may also be influenced by the onset of puberty, leading to more risky behavior, as well as higher impact forces, during sporting activities. In a recent retrospective case study, Zimmerman et al also observed a peak incidence of facial fractures around the onset of puberty, with sports injuries being one of the attributable factors. This review subsequently noted that facial fracture cases in patients under 5 years of age were exceedingly rare. 7

A strong gender association was detected as well, as our data demonstrated that males accounted for 82.4% of cases of facial fracture secondary to sports injury. In the United States, young boys participate in organized sports more frequently than young girls; however, this alone does not likely explain this disparity. 7 Studies have consistently shown a male predilection for pediatric facial fracture, and a recent 10-year study examining pediatric facial fractures etiology found 84.4% of their patients to be male. 8 Gleisner and Mukerji identified a smaller gender gap when looking at all etiologies of pediatric facial fractures. Their results indicated that 60% of pediatric facial fractures were sustained by males, and that this difference was, in part, attributable to sports-related injuries. 9 An additional study also cited that young males tend to participate in more dangerous activities than young females which often includes more high-contact sports. 7 Further evaluation is necessary to determine the significance of these gender disparities, and determine if gender-specific prevention strategies are worthy of investigation.

### Injury Pattern and Concomitant Injuries

We identified the orbit, mandible, and nasal bones as the most frequently fractured bones in our pediatric cohort. These findings are consistent with previous studies examining sports-related injuries. 10 In this 2008 study, Alcala-Galiano et al suggest that the progressive decrease in the skull-to-face ratio during adolescent development may cause a downward and forward projection of the face in pediatric patients, making the midface more susceptible to injury. The pediatric patient is also more susceptible to facial fractures due to less developed sinuses. As the sinuses mature they are able to absorb more impact and therefore transmit less force to surrounding facial bones. 10

In our cohort, facial injury was most commonly sustained during a baseball game, specifically, a direct blow to the face by a ball or bat. Each of these patients suffered an orbital fracture, and 60% also presented with an additional fracture of the nasal bone. While baseball may not be considered a contact sport, it is frequently indicated as one of the most common organized sports leading to injury and, specifically, facial fracture. 11 This is likely due to the lack of facial protection provided by commonly used baseball helmets. In a previous study, Danis et al examined the effect that utilization of helmets with facemasks on rates of baseball related facial injury. 12 They found a statistically significant decrease in facial injuries when these prevention measures were implemented. While this study was survey based, and injuries were not evaluated by a medical professional, this preliminary data suggested that addition of facemasks to baseball helmets may decrease the risk of facial injury without any significant athletic performance repercussions. Our findings, and the findings of these previous studies, indicate that further research is warranted to evaluate the efficacy of these types of prevention measures for children engaging in baseball related activities.

### Concomitant Injuries

Intracranial hemorrhage secondary to facial fracture may be fatal, and surviving patients may suffer long-term deficits. Three patients in our study were found to have suffered intracranial hemorrhage in addition to facial fracture. Each of these three patients sustained an orbital fracture, and two out of three also were found to have an additional facial fracture. A recent study examining intracranial hemorrhage in pediatric patients secondary to facial trauma found that fractures of the middle and upper face were more frequently associated with intracranial hemorrhage compared with those of the mandible. 4 While mandible fractures are common in adults, the most frequent factures identified in our pediatric cohort were of the middle and upper facial skeleton. While further large-scale investigation is required to assess the significance of this finding, it suggests that pediatric patients may be at greater risk of this potentially deadly complication.

In our cohort, close to 12% of patients presented with skull fracture and 23.5% suffered traumatic brain injury. As previously mentioned, the skull-to-face ratio decreases during development of the pediatric skeleton, leading to a downward and forward projection of the face. In younger pediatric patients, before this ratio decreases, skull fractures are more prevalent, as the bones of the skull may absorb a greater percentage of the impact in the setting of facial trauma. 10 This is another factor that should be taken in to consideration when designing protective gear for children participating in sports.

### Surgical Management

When managing pediatric facial fractures as compared with adults, growth potential must be taken in to consideration. While closed reduction and more conservative management may be associated with lower rates of postprocedure complications, the risk of long-term skeletal deformities must be considered. 10 13 Over half of the patients in our cohort were managed through surgical intervention with open reduction and internal fixation (ORIF). The most frequent method of fixation was implementation of titanium plates. In addition to titanium, bioabsorbable polymers have also gained popularity in recent years. 13 Further investigation into long-term outcomes is needed to assess the benefit of these new technologies.

## Conclusion

Sports related activities offer many physical and social benefits for developing adolescents, but it is imperative that these activities are safe for children to participate in. Due to developing facial anatomy and facial skeletal proportions, pediatric patients may be at increased risk for facial injury and fracture during sporting activities. In this cohort, we observed significant gender and age disparities. We also observed a relationship between specific sports, like baseball, and fractures of the middle and upper facial bones. Concomitant injuries were commonly identified in these patients, some as serious as intracranial hemorrhage and traumatic brain injury. These factors must be considered as future prevention strategies and protective equipment is developed.
